# An augmented reality approach for ADL support in Alzheimer’s disease: a crossover trial

**DOI:** 10.1186/s12984-019-0530-z

**Published:** 2019-06-03

**Authors:** Nina Rohrbach, Philipp Gulde, Alan Robert Armstrong, Linda Hartig, Anas Abdelrazeq, Stefan Schröder, Johanne Neuse, Timo Grimmer, Janine Diehl-Schmid, Joachim Hermsdörfer

**Affiliations:** 1 Chair of Human Movement Science, Department of Sport and Health Sciences, Technical University of Munich, Munich, Germany; 2 Cybernetics Lab IMA & IfU in Aachen, RWTH Aachen University, Aachen, Germany; 3 Department of Psychiatry and Psychotherapy, Klinikum rechts der Isar, Technical University of Munich, School of Medicine, Munich, Germany

**Keywords:** ADL, Augmented reality, Alzheimer’s disease, Usability, Assistive technology, Mixed methods

## Abstract

**Background:**

Dementia of the Alzheimer’s type can impair the performance of activities of daily living and therefore severely impact independent living. Assistive technologies can support such patients when carrying out daily tasks.

**Methods:**

In this crossover study, we used an augmented reality approach using a Microsoft HoloLens to support patients in a tea making task. During task execution, subjects received three-dimensional dynamic holograms of the sub-steps necessary to complete the task. Ten patients suffering from Alzheimer’s disease were tested and post-hoc semi-structured interviews were conducted to assess usability.

**Results:**

The patients committed errors when executing the task with and without holographic assistance. No differences in success rates or error frequencies were observed (p_success_ = .250, p_errors_ = .887). Patients revealed prolonged trial durations (Glass’ Δ = 1.475) when wearing the augmented reality headset. A model of multiple linear regression (R^2^_adjusted_ = .958) revealed an influence of the errors in the control condition and a moderation by the errors in the experimental condition. Patients with more severe problems in the natural performance of the task showed lower increases in trial durations when wearing the HoloLens.

**Conclusions:**

We assume that the application was a secondary task requesting its own resources and impairing performance on its own. The regression suggests however that the given assistance was compensating these additional costs in patients with stronger needs of support. Interview data on usability revealed an overall positive feedback towards the application although the hardware was considered uncomfortable and too large. We conclude that the approach proved feasible and the acceptability was overall high, although advances in hardware and the patient-interface are necessary to assist patients suffering from Alzheimer’s disease in daily activities.

**Trial registration:**

DRKS, DRKS00014870. Registered 11 June 2018 - Retrospectively registered, TrialID = DRKS00014870.

## Background

Cognitive deterioration in patients with dementia, especially of the Alzheimer’s type (AD), is known to negatively influence complex activities of daily living (ADL), such as shopping, navigating routines or preparing drinks and food [1–4]. Underlying factors can be loss of focus and memory function [5, 6] as well as signs of apraxia and action disorganization syndrome [7, 8]. The resulting ADL capacity can prohibit or limit independent living. So far, support is given by relatives and nursing services. The load and the cost of time and money are substantial for the patients and their relatives. While there is currently no cure for AD, a range of electronic devices to assist people with dementia has been developed [9, 10]. Augmented reality (AR) applications are a new possible approach to tackle these problems. AR can offer non-obtrusive guidance in everyday live.

Research shows that neurological patients are open-minded and have a positive attitude towards assistive technology to remain independent [11, 12]. The inclusion of the target group is thought to be crucial in the development process for the usability of the assistive technology end-product [12]. Usability can be defined as “the extent to which a product can be used by specified users to achieve specified goals with effectiveness, efficiency and satisfaction in a specified context of use” [13]. In this crossover study, we examined the feasibility and usability of the AR approach using a head mounted Microsoft HoloLens (Microsoft Cooperation) to support patients with Alzheimer’s disease in the execution of the ADL of tea making. Additional to performance parameters, we applied semi-structured interviews to involve the end users opinion.

## Methods

### Analytic approach

To examine the usability of AR guidance during the ADL of tea making in patients with AD, we applied a mixed method design [14] to obtain quantitatively abundant performance data by running a crossover study as well as the patients´ individual experiences conducting semi-structured interviews. Within the crossover study, the same ADL task (tea making) was performed in two conditions, one being the control condition (natural tea making), and the other being the experimental condition (AR-supported tea making). This design is useful because it allows a perfect match of subject characteristics as measurements of the same participants are compared. As our aim was to contextualize our quantitative findings using qualitative data, the reported results primarily stem from our quantitative data, but parallel data analysis helped to complement our findings [14].

### Tea making task

The tea making task has been selected as an example of a relevant ADL task because it requires the ability to organize multi-step actions in a sequence of subtasks to achieve a goal, is highly relevant in many peoples life, and has been intensively studied in the literature in patients with brain damage, e.g. suffering from apraxia and action disorganization syndrome [15, 16].

### Hardware

The Microsoft HoloLens (Microsoft Cooperation) head mounted display was chosen as a state of the art technology that would enable freedom of movement for users while still possessing the ability to deliver support through its built in mixed reality technology.

### Software development

The AR application was developed within a user centered design approach consisting of four iterative cycles (March 2017 – December 2017) through collaboration between researchers, clinicians, patients and family members in the framework of the EU project “Therapy Lens” [17]. As recommended in the literature [12], testing in these predeployment phases took place in the patient’s daily living environment outside the lab to better understand the needs and make participants feel more comfortable and part of the design process. The given feedback of different stakeholders [18, 19] resulted in the design of a step by step guidance system for a multi-step ADL task (tea making), incorporating audio-visual cues for each step, namely asking to:

1. Fill water into the kettle
2. Switch the kettle on
3. Add a tea bag to the mug
4. Wait for the water to boil
5. Pour the hot water into the mug
6. Remove the tea bag
7. Task is finished

**Fig. 1:**
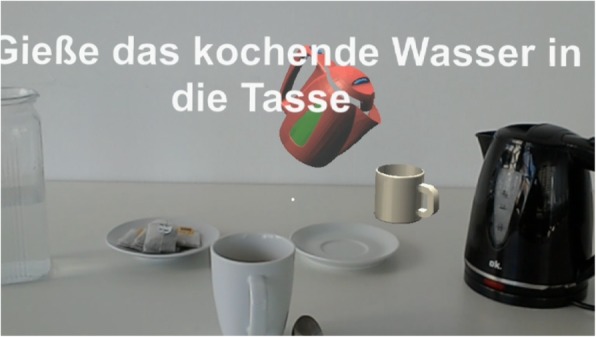
Display of holographic cues presented by the Therapy Lens application on a Microsoft HoloLens. The subtitle and holograms indicate the first step in the tea making task of pouring the heated water into the mug (“Gieße das kochende Wasser in die Tasse”). The red kettle and the small white mug (on the right) are both holographic objects

Cues are given by a young female voice instructing the next step (including a displayed subtitle) and a holographic simulation of the corresponding step (Fig. 1).

Interactions are possible with the interface using three different control strategies, namely hand gestures, a clicker (similar to a computer mouse) or voice control. Given the novel nature of the device and the feedback during our development process, we decided to simplify the interaction to only one control strategy. The pilot interviews revealed a clear preference for speech recognition as the primary control strategy because of being the most intuitive allowing the hands free to interact [19]. Thus, after the completion of each step the patient proceeds to the next step by the voice command “weiter” (German for “next”, recognized by a speech recognizer in the application). The application remains in the current step if the command (“weiter”) is not given. The Therapy Lens application was developed in Unity 3D 2017.1.0 with the compatible HoloLens Tool Kit. The final demo of the used prototype was published and is freely available on the Microsoft Store since February 2018 under the name Therapy Lens [17].

### Participants

This crossover study took place at the Center for Cognitive Disorders at the Department of Psychiatry and Cognitive Rehabilitation of the Klinkum rechts der Isar, Technical University of Munich in Germany, from January to March 2018. Participants were recruited, based on the following eligibility criteria: adult patients with diagnosed dementia of the Alzheimer’s type, a normal or corrected-to-normal vision, and sufficient cognitive ability to understand and follow the task instructions. The sample consisted of 10 patients (71.8 ± 11.1a; 7 male, 3 female) suffering from mild and moderate dementia of the Alzheimer’s type (Table 1).

**Table 1:** Details of the patient sample

Patient	Age [1a]	Sex	Diagnosis (ICD)	Education	MMSE	Order of conditions
01	64	M	F00, F32.0	Diploma	22	C-T
02	69	F	F00	Doctor	19	T-C
03	51	M	F00, F32.0	Diploma	27	C-T
04	78	F	F00, F32.0	School	24	C-T
05	84	M	F00	Diploma	18	C-T
06	81	M	F00	Apprenticeship	25	C-T
07	57	M	F00	Apprenticeship	21	T-C
08	80	M	F00	Apprenticeship	27	C-T
09	77	F	F00, F32.0	Apprenticeship	25	T-C
10	77	M	F00	Apprenticeship	19	T-C
*n = 10*	*71.8, ±11.1*	*7x male, 3x female*	*10x AD, 4x depression*	*1x doctor, 3x diploma, 5x apprenticeship, 1x school*	*22.7 ±3.4*	*5x C-T, 5x T-C*

*Legends: M male, F female, ICD international classification of diseases, AD Alzheimer’s disease, MMSE Mini Mental State Examination, C-T control – Therapy Lens, T-C Therapy Lens - control; F00 Dementia of the Alzheimer’s type; F32.0 mild depression*

### Ethical considerations

Ethical approval in accordance to the declaration of Helsinki was obtained from the Ethics Committee of the Medical Faculty of the Technical University of Munich (reference number 175/17 S). All participants gave written informed consent.

### Usability testing

**Fig. 2:**
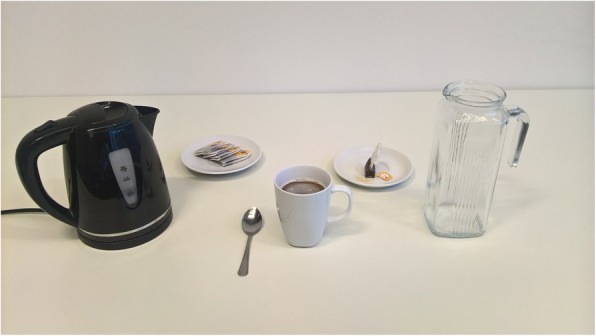
Picture of the end product (hot cup of tea) shown to all patients prior to each trial. Objects from left to right: Kettle, tea bags on a saucer, spoon, mug, saucer for used tea bags, and water container (filled with 500ml of room temperature water at the start of each trial)

Testing included the preparation of a hot cup of tea. Tea making was carried out twice for each of the conditions. These were the natural condition (control condition) and trials guided by an augmented reality application (Therapy Lens condition) for the Microsoft HoloLens. The first trial in both conditions was always a familiarization trial and not scored. Based on our experiences made in the previous developmental stages, we put emphasis on the correct fitting of the glasses as people who never experienced the HoloLens before tend to need more time for proper adjustment. As we were interested in the intuitive handling of the application’s current form, the orientation with the device and its usage focused on a brief introduction to its basic functioning and control via voice command. In the natural condition patients were asked to prepare a cup of tea in a natural way, as if they were at home, with no emphasis on speed or accuracy, while in the guided condition patients were asked to follow the instructions given by the system step by step. Prior to all trials a DIN A4 picture of the end product (hot cup of tea) was shown to the patients (Fig. 2). The assignment to the orders of conditions were pseudo-randomly set prior to the first patient contact. Blinding of patients or researchers was not possible due to the device being used (AR glasses). The usability of the system was further assessed by video observations of dementia patients using the AR application and the conduction of semi-structured interviews.

**Fig. 3:**
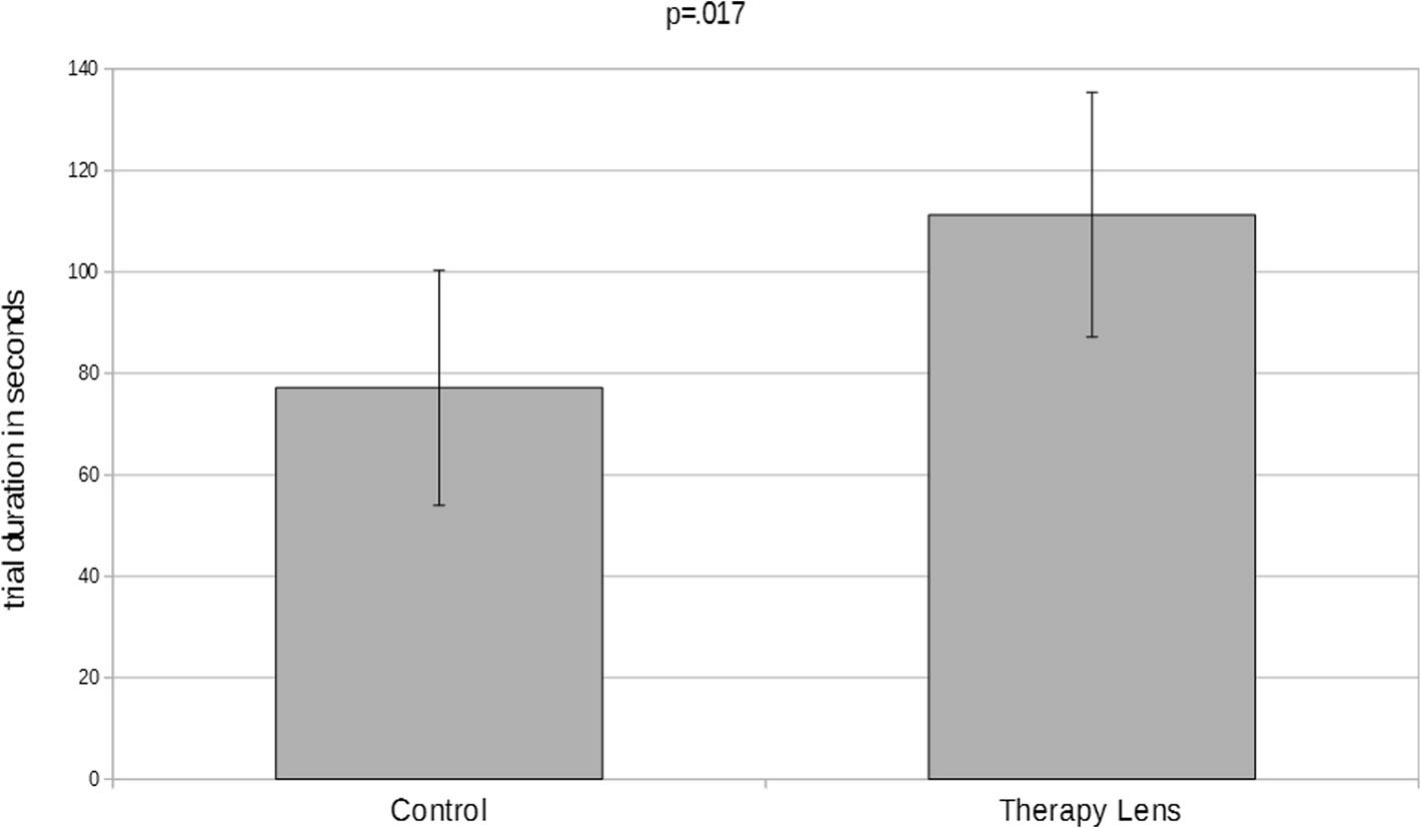
Means and standard deviations of the trial durations in seconds for the two conditions: control and Therapy Lens

**Fig. 4:**
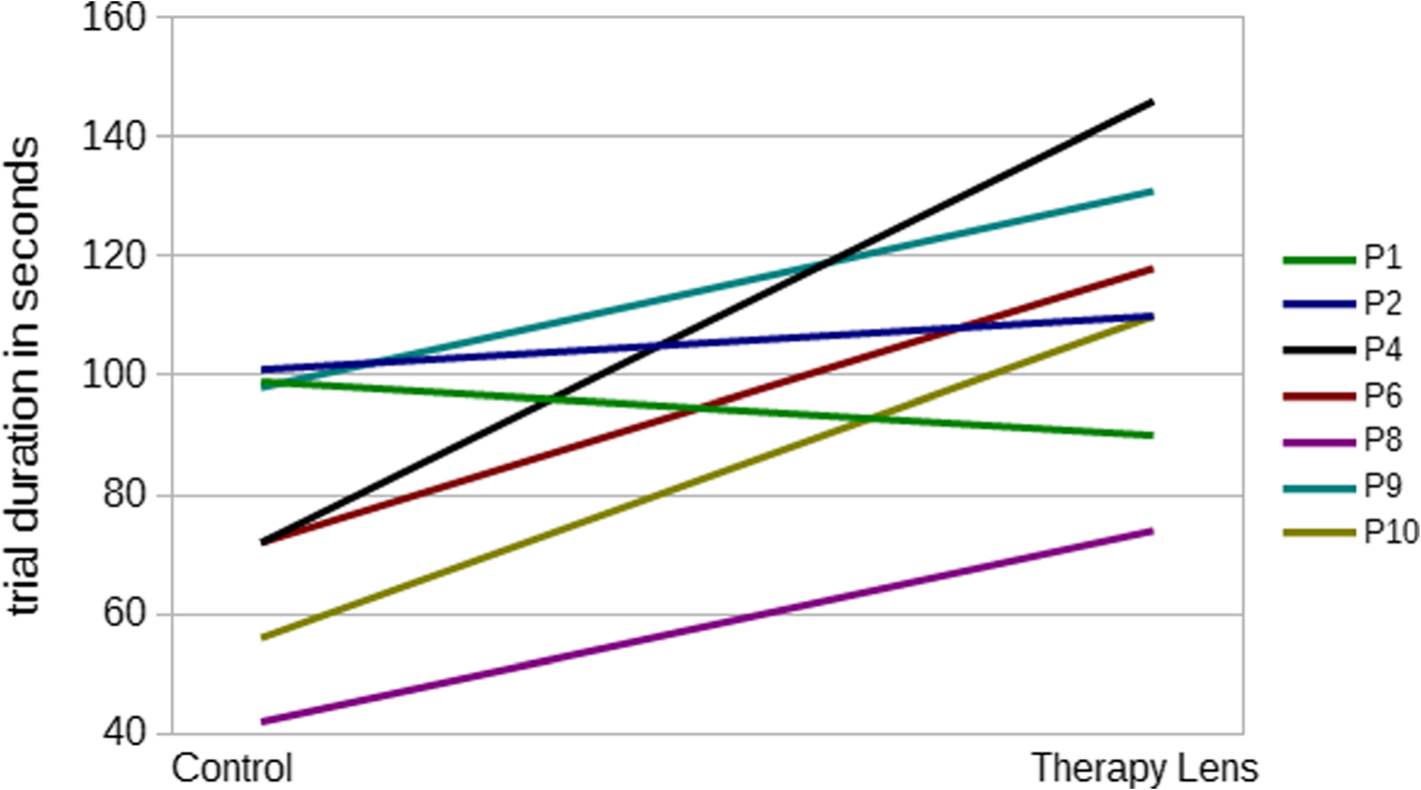
Change of trial durations between the control and the Therapy Lens condition for the seven patients with successful trials in both conditions

**Fig. 5:**
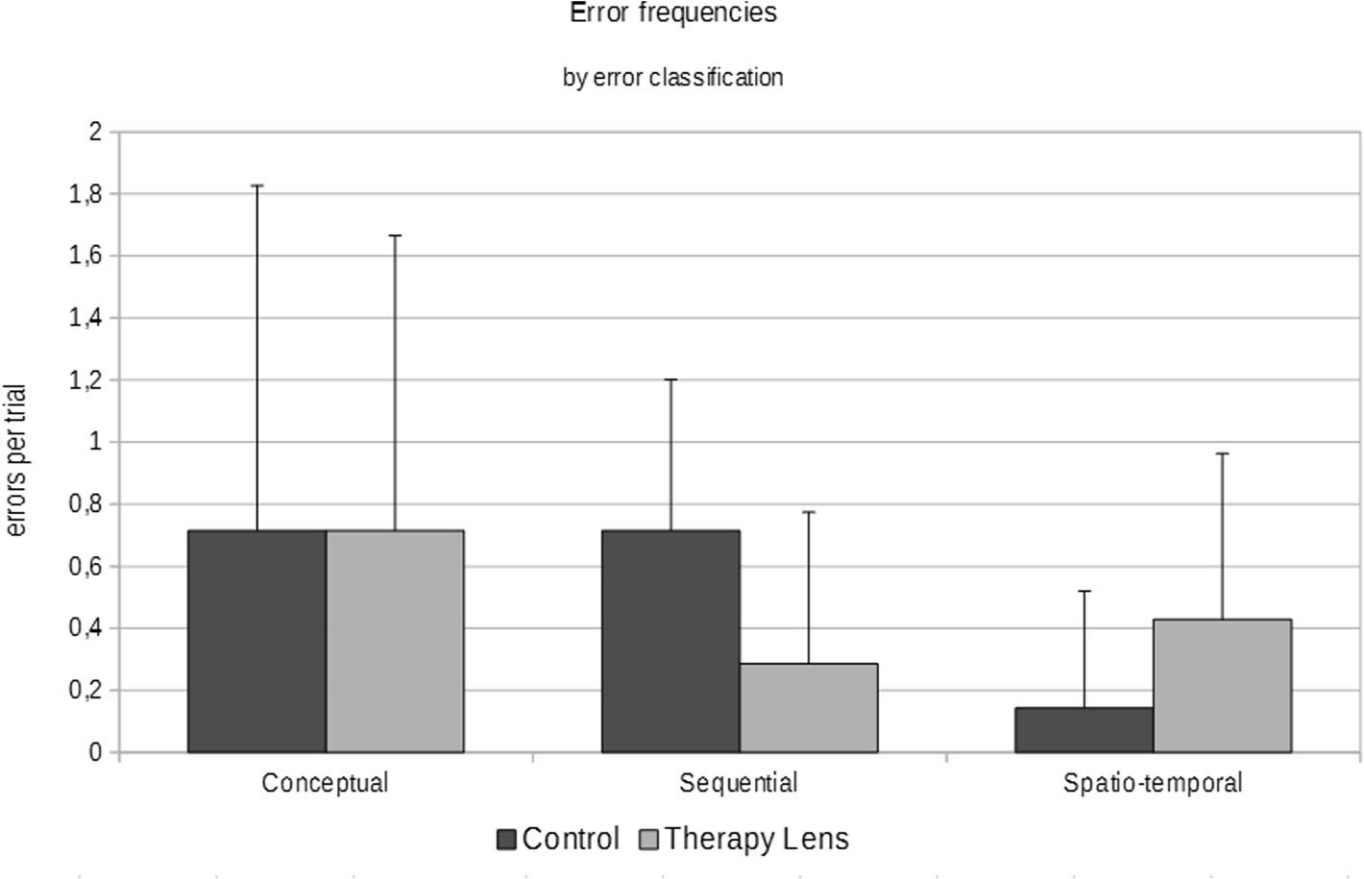
Error frequencies by error classification in the control and Therapy Lens condition in successful trials

**Fig. 6:**
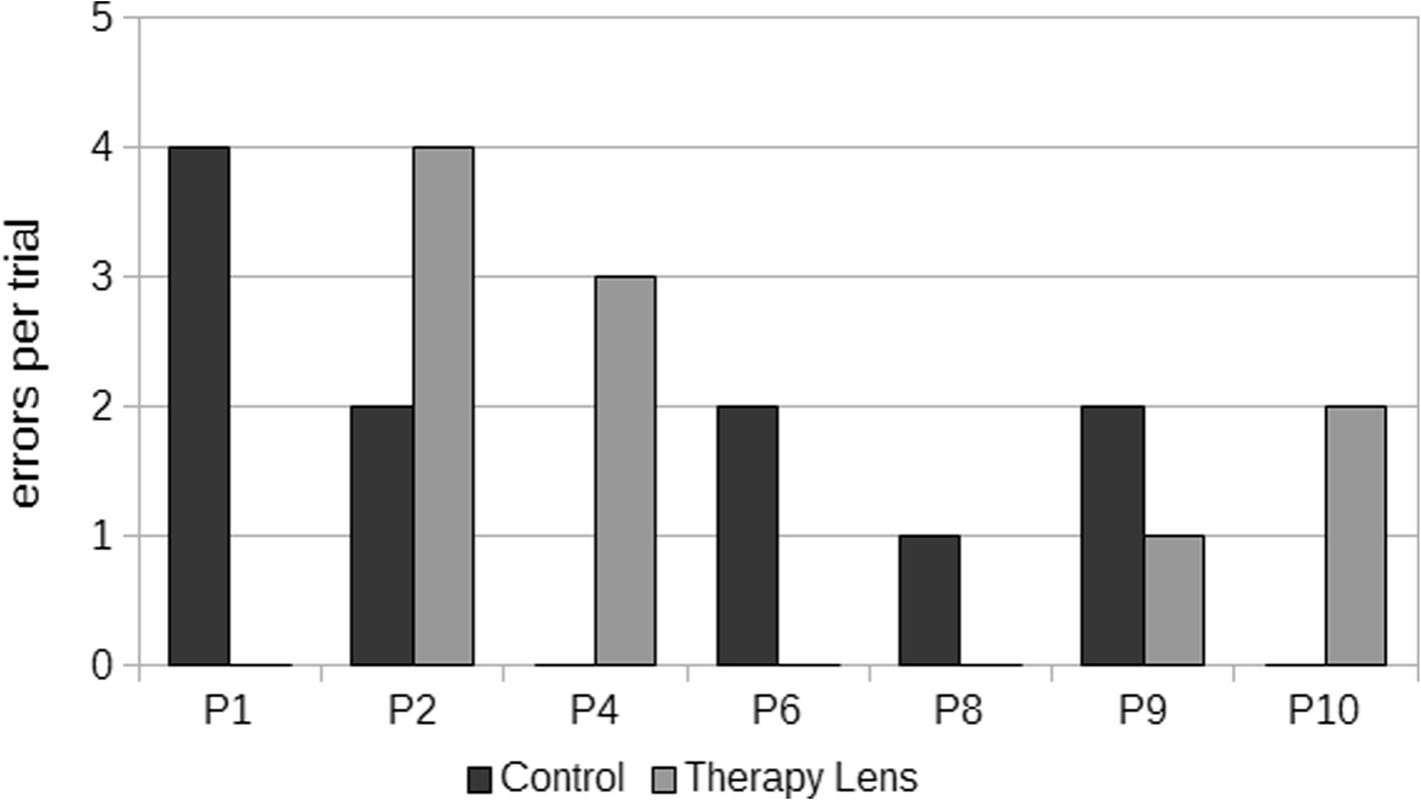
Error occurrences in the control and the Therapy Lens condition for the seven patients with successful trials in both conditions

**Fig. 7:**
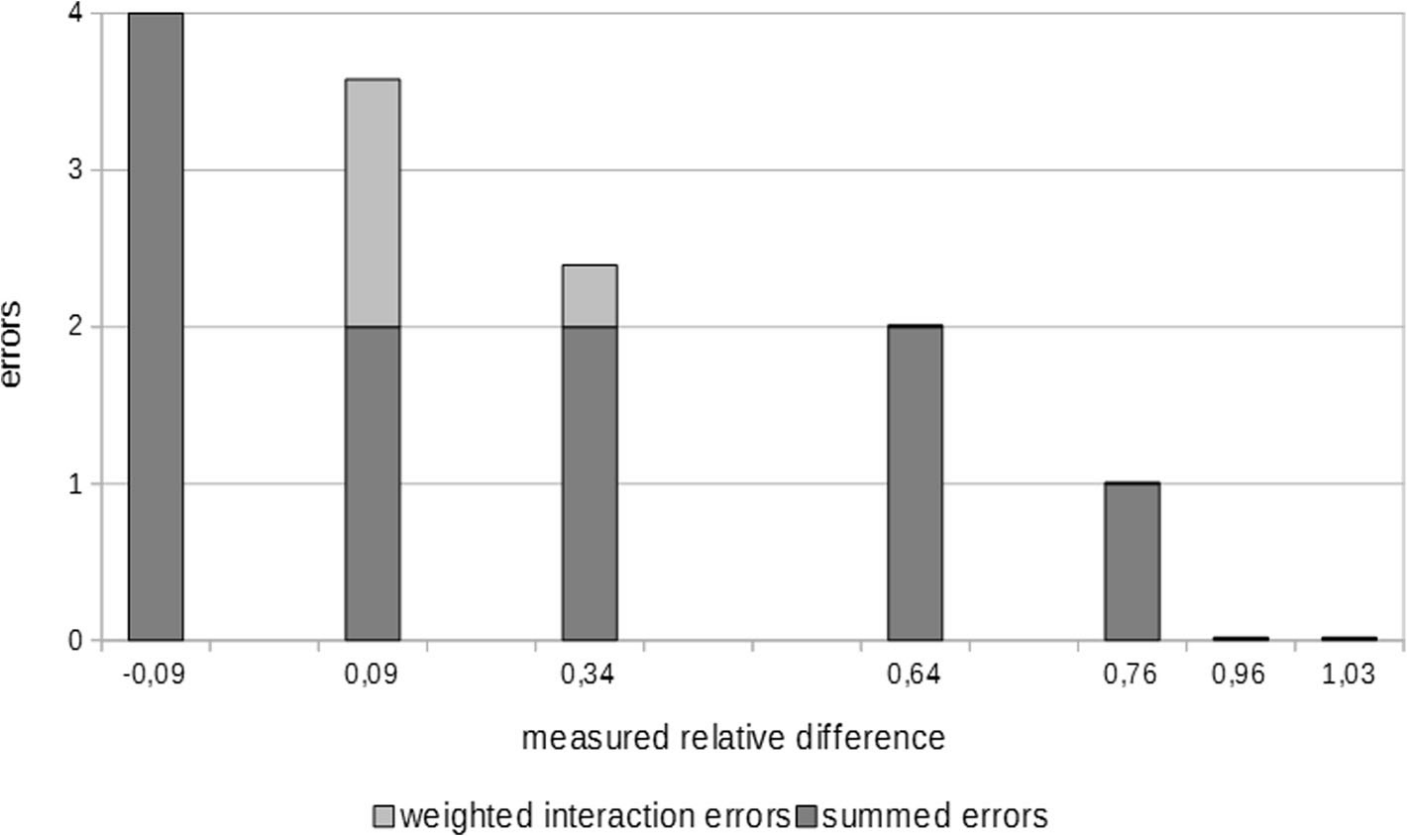
Association of measured relative difference and error according to the model of multiple linear regression (R^2^_adjusted_ = .958, *p* < .01). The interaction errors (control condition x Therapy Lens condition) are weighted based on the model’s coefficients. Each bar represents one of the patients

### Performance analysis

Used parameters for the performance analysis were the **trial durations** for the second trial of each condition (inactive waiting time for the water to boil was excluded), the **relative difference between the trial durations** of successful control and Therapy Lens trials, the **success** of achieving the task goal (hot cup of tea), the **age**, the **order of conditions**, and the **MMSE score**. Trial duration has been shown to be a valid marker of performance in the chosen task [4].

1 $$ relative\kern0.17em difference=\frac{Therapy\;{Lens}_{trial\kern0.17em duration}-{Control}_{trial\kern0.17em duration}}{Control_{trial\kern0.17em duration}} $$

Further, an error analysis, based on video recordings was performed and **errors** were assigned to one of three error categories, namely: spatio-temporal, conceptual, and sequential [15]. Commonly observed ADL difficulties, as errors in the execution of the task (e.g. dropping an item) or the mislocation of an object (e.g. pouring water onto the table rather than into the glass) are scored as spatio-temporal errors. An example for a typical conceptual error is an action that is carried out, but not in an appropriate way (e.g. failing to open the kettle). Often observed sequencing errors include behaviors like performing an action much later than usual (e.g. switching the kettle on after preparing the cup of tea) or unintentionally omitting a step (e.g. turning the kettle on without having inserted water) [15].

### Statistical analysis

The statistical analyses of the performance data included a McNemar test for paired samples to compare the number of successful trials between the conditions (control and Therapy Lens). For the parametric tests, only trial durations of pairs of successful trials were used for the analyses. Further, a Shapiro-Wilk test was applied to test for normal distribution of the trial durations. Then a repeated measures ANOVA was computed to compare the trial durations of both conditions. Finally, a repeated measures ANOVA with the between-subject factor order of conditions and the covariates age and MMSE score was used to compare the trial durations with respect to effects of order, age, or mental capacity. Additionally, a multiple linear regression (MLR) was run to model the relative differences in trial durations of successful trial pairs based the error metrics, age, MMSE, and the order of conditions. Effect sizes were Glass’ Δ for the condition comparison. Variance inflation factor was set to < 5.00. α was set to .05.

### Qualitative content analysis

The interviews lasted approximately 15 min, and were held in German language. They were based on an interview guide that consisted of general open questions regarding patients’ experiences with the AR system and specific open questions on satisfaction with the hardware and the multi-dimensional support given by the system (Table 2). Interviews were audio recorded, transcribed word-for-word according to specified guidelines [20] using the software f4/f5transkript (dr. dressing & pehl GmbH) and pseudonymized. Interview data were analyzed using the structuring qualitative content analysis described by Kuckartz [20]. Main categories were formed a priori based on the lead questions and the literature [12] (Table 2).

**Table 2:** Three major codes affecting the usability

Code	Definition	Examples
Hardware	Hardware related factors influencing the user-friendliness and satisfaction	• Wearing comfort & design
		• Estimated duration of daily use
Software	Software related aspects influencing the user-friendliness and satisfaction	• Layout & design of cues
		• Structure
		• Functioning (command)
Acceptability	Reactions and emotions to the system Factors affecting the willingness to use the system	• Meaningfulness
		• Capabilities & Control
		• Effect of novelty

The interview data were coded and analyzed using the software MAXQDA Analytics Pro 2018 (Release 18.0.0, VERBI GmbH) by one person (NR). Based on systematically prepared thematic summaries, common themes were extracted, analyzed, and the meanings discussed with a second researcher (LH). Three major categories describing the usability of the AR application are presented in Table 2. The code “hardware” was assigned when device related barriers or facilitators were highlighted to affect the systems usability, e.g., the general wearing comfort and design of the hardware or the estimated acceptable duration to wear it during the day. The code “software” captured software related aspects influencing the usability, e.g., the design and the structure of the different presented cues or the reliability of the technology. The code “acceptability” served to capture the user’s reactions to the system, i.e., emotions, and the acceptability based on a patient’s capability to understand and use it. It includes factors affecting their willingness to use the system, e.g., the lack of a consumer’s perceived benefit from using the system (meaningfulness), or positive and negative effects of a novel and unknown technology.

## Results

### Performance analysis

The average time to successfully perform the task was 77.14 s ±23.15 s in the control condition and 111.29s ± 24.10 s in the Therapy Lens condition (Table 3, Figs. 3 & 4). In the Therapy Lens condition three patients (P03, P05, P07) failed to successfully execute the task, while all patients were able to achieve the goal in the control condition (not statistically different, McNemar *p* = .250). The trial durations in both conditions were normal distributed (Shapiro-Wilk Control: *p* = .267, Therapy Lens: *p* = .955). A repeated measures ANOVA revealed a significant difference in trial durations between the conditions (*p* = .017, partial Eta^2^ = .638, Glass’ Δ = 1.475). When including the order of conditions as a between-subject factor and age and MMSE as covariates, the resulting repeated measure ANOVA showed no effect of condition (*p* = .199), any interaction, or significance for the order of conditions (*p* = .617), the age (*p* = .691), and the MMSE score (*p* = .867).

**Table 3:** Means, standard deviations, significance levels, effect sizes for the used parameters in the two conditions Therapy Lens and control

	Trial durations†	Relative difference†	Successful/failed trials	Summed errors†	Conceptual errors†	Sequential errors†	Spatio-temporal errors†
Control condition	77.14 s	.53 ± .43	10/0	1.57	.71	.71	.14
	±23.15 s			±1.40	±1.11	±.49	±.38
Therapy Lens condition	111.29 s		7/3	1.43	.71	.29	.43
	±24.10s			±1.62	±.95	±.49	±.53
Significance	p = .017 Glass’ Δ = 1.475	–	p = .250	*p* = .887	*p* = 1.000	*p* = .078	*p* = .356

Legends*: †Based on pairs of successful trials*

The error analyses showed no significant differences for neither the summed up errors (*p* = .887) or the different error categories (conceptual: *p* = 1.000 , sequential: *p* = .078, spatio-temporal: *p* = .356) (Table 3, Figs. 5 & 6).

Multiple linear regression revealed an impact of the summed up errors in the control condition, moderated by the summed up errors in the Therapy Lens condition (interaction term). The resulting R^2^_adjusted_ was .958 (*p* < .01). The β-weights were Errors_control_ = −.858 (*p* < .01) and interaction term Errors_control_ x Errors_Therapy Lens_ = −.361 (*p* = .01). The moderation reduced the β-weight of Errors_control_ from −.919 to −.858. All means and standard deviations are shown in Table 3. The results of the MLR are displayed in Fig. 7.

2 $$ predicted\kern0.17em relative\kern0.17em difference=1.023-.264\ast {Errors}_{control}-.052\ast Interaction\;{Term}_{Errors\kern0.17em Therapy\kern0.17em Lens\;x\kern0.24em control} $$

### Unsuccessful trials

Three patients failed to successfully use the Therapy Lens application, ergo were not able to achieve the task goal (hot cup of tea). Video analyses revealed that one patient (P05) failed to proceed further than step 1 (“Fill water into the kettle”), due to the inability to open the kettle’s lid. Two patients (P03, P07) did not proceed beyond the second step (“Switch the kettle on”). One patient (P07) first tried to use the switch to open the kettle and therefore switched the kettle off after closing the lid. The other patient (P03) removed the kettle from its base when filling in the water and did not place it back on the basis (precluding power supply). Thus, in both cases the water did not start boiling and step 4 (“Wait for the water to boil”) was therefore not achieved.

### Interview data

The analysis of the semi-structured interviews revealed a range of opinions on the presented ADL support system. We identified three major categories from the content analysis of the interview data affecting the usability of the system: hardware and software related issues and the acceptability (Table 2).

### Hardware

According to the interviews, most of the patients (70%) could imagine to wear the AR headset between 15 and 60 min a day before they would need a break, with a maximal mentioned duration from “less than one minute” (10%; P05) to “up to several hours including breaks” (20%; P06, P10). The core aspect criticized by the patients referred to the hardware related wearing comfort. While two participants valued the device as relatively light weighted (20%), six participants (60%) criticized it by describing it as: “too big”, “bulky”, “impractical”, “heavy”, “obstructive”, or “monstrous”, which was influencing the extent to which the application would be used, as communicated by one patient (P04):

“I liked it, but I do not want to wear it. [ …] Because that would bother me, because it’s just such a big thing. [ …] It’s great, but I do not want it (laughs). It’s great because you can read it nicely in there. It is very clear, you can read it clearly, clearly big. [ …] But that’s such a big thing. It’s just too big.”

### Software

The majority of examined patients (90%) was able to control the system using the required voice command “weiter”. 40% of patients highlighted the well reacting speech function. However, patients occasionally needed extended periods of time to remember the correct command, or, in other cases, patients automatically carried out the task without making use of the speech command or were passively waiting for more instructions after a step was finished. Thus, the fixed sequence of actions (step 1–7) was confusing for some participants who only used it partially. For instance, P03 proceeded with step 3 (“Add a tea bag to the mug”) before giving the speech command to trigger this specific step. The patient was asking for more information and feedback, e.g., about the total amount of steps needed to fulfill the task. At the end, the patient failed to execute the task due to the inability to boil the water. The patient expressed the situation as follows (P03):

“Then the waiting until the next step [ …] and then the question how long does it take? [ …] And then wait until someone says it is enough? Or does it proceed on its own? [ …] When is this stupid thing (kettle) finally boiling or will there come several steps [ …] and one step with the tea bag was not right. First came … , that was reversed, I think, there was somehow a reversal of the order.”

The opinion on the multi-dimensional cues (audio, subtitle, and holograms) differed between participants. While some recommended keeping text information because of potential hearing problems (40%), others were in favor of audio support due to potential visual impairments (50%). Two patients positively mentioned the “clear readability” and the “pleasant voice” (20%). Most patients seemed to appreciate the combination of several dimensions to “avoid misunderstandings” (P09). 40% of patients were highlighting the holographic animations because of being “easier to store” (P01). One patient emphasized (P03):

“[ …] But a picture is worth a thousand words. This is already clear.”

No negative opinion was given towards the holographic animations, but two patients were not able to remember them at all (20%; P05, P08).

### Acceptability

The users judged their experiences differently. One patient described the experience as unusual and very new, since the patient has never used such a device before, and that there was a need for more time and interaction to orientate and to build an opinion on whether it is useful for the patient or others (P07). In another case, the cueing system caught a patient’s attention who described it as (P09):

“[ …] very interesting [ …], so I became curious.”

While some patients stated an added value for daily task support, e.g., one saying that with the help of the AR support the patient “would not forget anything” (P04), there were others who were not willing to use the application (P05), did not fully understand the concept (P02), or were questioning the application because of not seeing its meaningfulness due denying their diagnosis or need for support (P09):

“I do not think I need help at home.”

## Discussion

In this study we introduced an augmented reality application via a headset to patients with Alzheimer’s disease in order to support them during the performance of the activity of daily living of making a cup of tea. Our analyses revealed that the introduction of the Therapy Lens application had no clear positive effect on the patients’ performance. Errors during task execution did not change significantly, although a trend (*p* = 0.078) towards less sequencing errors in TL could be observed (Table 3, Fig. 5). Further, the duration of task execution actually increased in TL (Table 3, Figs. 4 & 5). We could not find influences of the order of conditions, the age, or the MMSE score on the prolonged trial duration. Apparently, neither the age nor the mental capacity are good predictors, if and to what degree the application of Therapy Lens is detrimental. Data from semi-structured interviews on the usability of the AR headset revealed an overall positive experience, although the hardware was still considered as uncomfortable and too large (e.g., “bulky”). Even though the reliability of answers of AD patients is sometimes questionable [21], made observations and the patients’ opinion in this study allow us to gain a better understanding in how AR applications can assist daily life activities in AD patients.

In the control condition participants were asked to perform the task of making a cup of tea in a natural way, without emphasis on speed or accuracy. From a qualitative perspective (see Software), we assume the longer trial durations in the Therapy Lens condition to be partially based on the following factors:

1. Patients were following a predefined order of steps not allowing for a simultaneous execution of steps.
2. Patients awaiting and perceiving the cues before performing the current step and pausing after the execution of a step and awaiting further feedback or instruction.
3. Patients not immediately remembering the appropriate voice command.

Quantitative data allowed modeling of the relative differences of trial durations between the two conditions. The resulting MLR revealed that increments of trial durations in the Therapy Lens condition were strongly dependent on the performance, in terms of errors, in the control condition, whereat increased error occurrences where associated with smaller relative differences. This was moderated by the error frequencies in the Therapy Lens condition. Taken together, the less support would have been needed in the control condition the worse was the application of the Therapy Lens for the ADL performance in terms of duration. This was to a small part caused by problems interacting with the augmented reality headset (βΔ Errors_control_ = .061), but mainly by the burden of a secondary task being partially compensated by the support of the application in patients with ADL impairments.

The acceptance of assistive technologies is expected to vary during the course of dementia, i.e., acceptance can improve when symptoms start to threaten the independence of the patient [12]. Having this in mind and supported by the MLR, we suggest that the presented “step by step” approach may be most beneficial for more severe affected patients. However, when targeting this patient group one has to consider the possible resistance by the users when denying their diagnosis, as often observed in people with dementia [12] and depicted by our qualitative data (see Acceptability). For instance, one of the patients (P05) had a very strong reluctance in accepting the AR approach, stating that he does not want to use it. He also failed to complete the task in the AR condition because of not being able to open the lid of the kettle even though he managed this step before in the natural condition. His denial might have negatively influenced his performance.

Another important aspect to consider is that neurodegenerative changes caused by dementia can even make using mainstream devices problematic for some people with dementia. Further, the reduced ability for new learning in dementia patients may impact actual usage of a novel technology [12]. Herein, we confronted patients with a new technology that we introduced only by a short familiarization trial. The AR application required higher cognitive demands when processing the augmented cues and controlling the new device while performing a complex multi-step task. While we tried to keep the handling of the device as simple as possible by using speech control that requires only one word, both, the application of the command and the integration of the predefined “step by step” guidance into an often performed task appeared challenging for patients. This could potentially be compensated for by a longer familiarization period or practice sessions. Additional feedback given by the system, e.g. a holographic timer providing information on the brewing time as demanded by one patient (P03) or a reminder function after a certain time of pausing in the case a patient is losing focus, might potentially also support usage.

Including participants early in the development of assistive technology is recommended [12]. Indeed, when qualitatively reviewing the video recordings and analyzing the interview data, it became obvious that reasons for failure or longer trial durations seemed to be largely due to a lack of intuitiveness. The experienced malfunctioning of a technical device is potentially frustrating the user, thus, influencing the willingness to use the application. Consequently, to enhance the final acceptance of such an application it is vital to integrate the users’ feedback into future development. Apparently, as soon as technical support is given, users trust the system’s instructions with the given risk of over-reliance. In the tested system, the implemented number of seven steps was insufficient as patients got confused due to missing details. For instance, another reason why one of the patients (P03) failed in the AR condition was due to a missing cue between the first and the second step (i.e. placing the kettle to its base after filling in the water to allow boiling). Even though he was wondering why the kettle was not starting to boil the water, he was not able to solve the problem himself, but relying on the given instructions instead. We therefore suggest increasing the amount of support by integrating a higher quantity and more detailed steps (i.e. opening and closing the lid of the kettle) to allow for unrestrictive and straightforward guidance. Besides the mentioned discomfort related to the uncomfortable and large hardware, we did not observe any adverse events; like motion sickness or headaches.

Addressing the heterogeneous needs of persons with dementia is a well-known challenge [12]. Based on our study, patients value the integration of multiple cues (audio, text, holograms). The potential of holographic animations to support ADL tasks was supported by patients stating that their attention was caught and their interest awakened. However, not all patients noticed or remembered the holograms. We hypothesize that the simultaneous presentation of multiple cues was overwhelming, thus some information was masked out. In future trials, different cues (e.g., number and mode of presentation) should be evaluated against each other to investigate the most beneficial way of augmenting feedback to the real world environment in people with limited cognitive performance, although the used multi-modal approach ensured reliable cueing when dealing with comorbidities like partial loss of vision or hearing problems.

## Conclusions

In conclusion, the prolonged duration of the experimental condition may be interpreted as an indicator of impaired performance of the ADL task, as a result of dealing with a secondary task (AR application). So far, the constraints, i.e., the unnatural interaction with the application and a drag of attention to the holograms from the real objects, preponderate the support in sequencing the task to a goal directed order of steps. Still, MLR revealed that in patients with more severely impaired performance dual task costs due to the application were almost balanced by the given support. Overall, the acceptability of the AR application appeared to be high, as a large part of participants revealed a positive attitude towards the system, although the hardware was considered the main impediment. This leads us to the conclusion that the paradigm of augmented support is generally working, but the implementation still needs an improved user-interface. Future hardware advances in AR will allow such applications to significantly assist patients with ADL impairments and promote independent living. The aim of the study was to test for usability and feasibility but also to provide directions for further improvements. To increase intuitiveness of our system, the next step will be to incorporate the obtained feedback in our future adjustments, followed by a postdeployment stage in close partnership with all potential end users, including clinicians and carers. Specifically, we will focus on the 1) optimization of cues by increasing the amount and details of the steps; 2) promotion of familiarization by incorporating a longer practice session; and 3) personalization by allowing the user to decide on the type of feedback (holographic animations and/or audio and/or text).

## Abbreviations

AD: Alzheimer’s disease
ADL: Activity/ies of daily living
AR: Augmented reality
C-T: Order of conditions: Control condition first
F / M: Female / Male
MLR: Model of multiple linear regression
MMSE: Mini Mental State Examination
T-C: Order of conditions: Therapy Lens condition first
